# Supplementary material for: Direct visualization of human myosin II force generation using DNA origami-based thick filaments
Source: Commun Biol. 2019 Nov 27;2:437. doi: 10.1038/s42003-019-0683-0 (PMC6881340; doi:10.1038/s42003-019-0683-0)
Supplement: Supplementary file 1 — Supplementary Information [file 42003_2019_683_MOESM1_ESM.pdf]

Supplementary Information

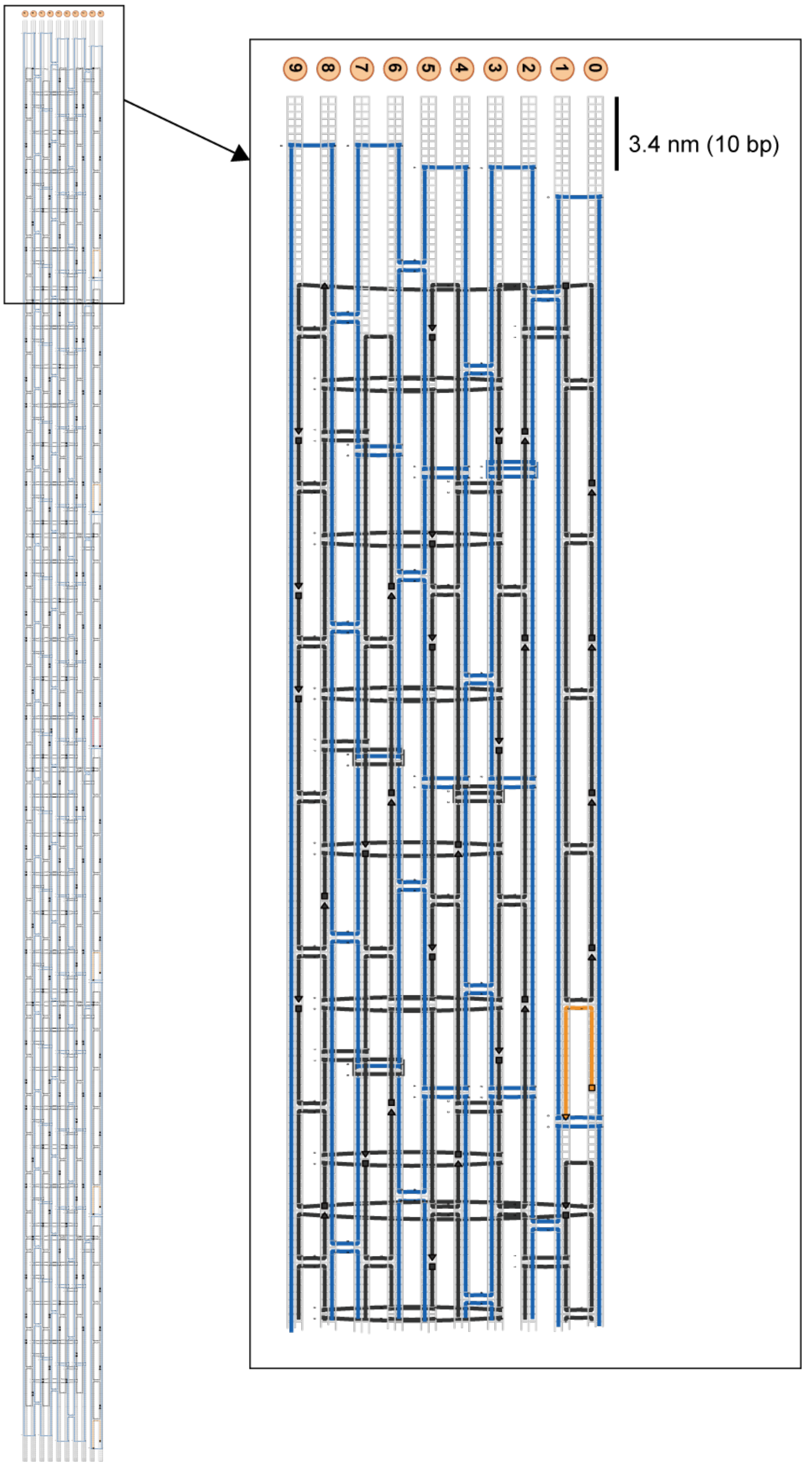

Supplementary Figure 1. Design of the DNA origami rod for the engineered thick filament. The scheme was produced by caDNAno2 software (https://cadnano.org/). Scaffold, core staples, handles for linking with myosin and the actin binding domain of α-actinin are shown in blue, black, red and orange, respectively. The boxed area is expanded on the right. Numbers in the circles at the top of the boxed area indicate the index of the double-stranded DNA in the DNA origami structure.

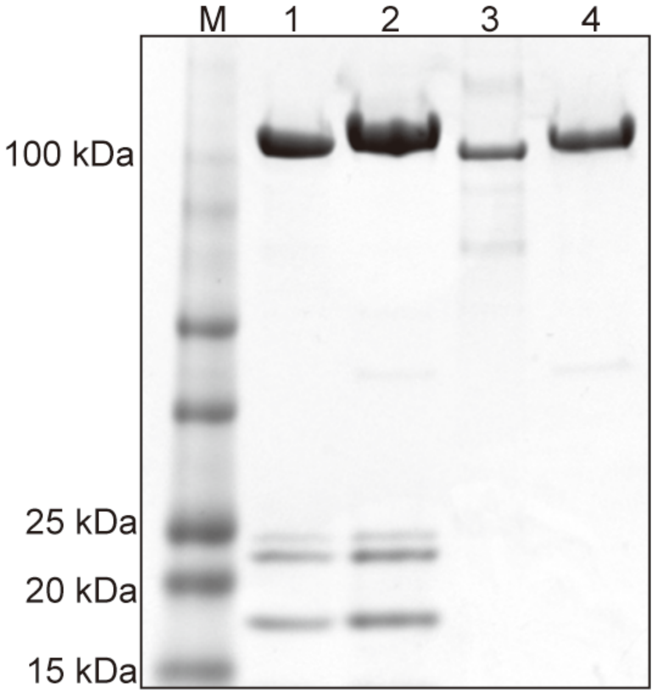

Supplementary Figure 2. SDS-PAGE gel of human myosin IIa S1 and lever-arm-less S1. Myosin S1 expresses light chains (15-25 kDa); lever-arm-less S1 does not. A band shift for heavy chains (~100 kDa) confirmed the labeling of oligonucleotides. M, marker; lane 1, myosin IIa S1; lane 2, myosin IIa S1 labeled with a 21 mer oligonucleotide; lane 3, lever-arm-less S1; lane 4, lever-arm-less S1 labeled with a 21 mer oligonucleotide.

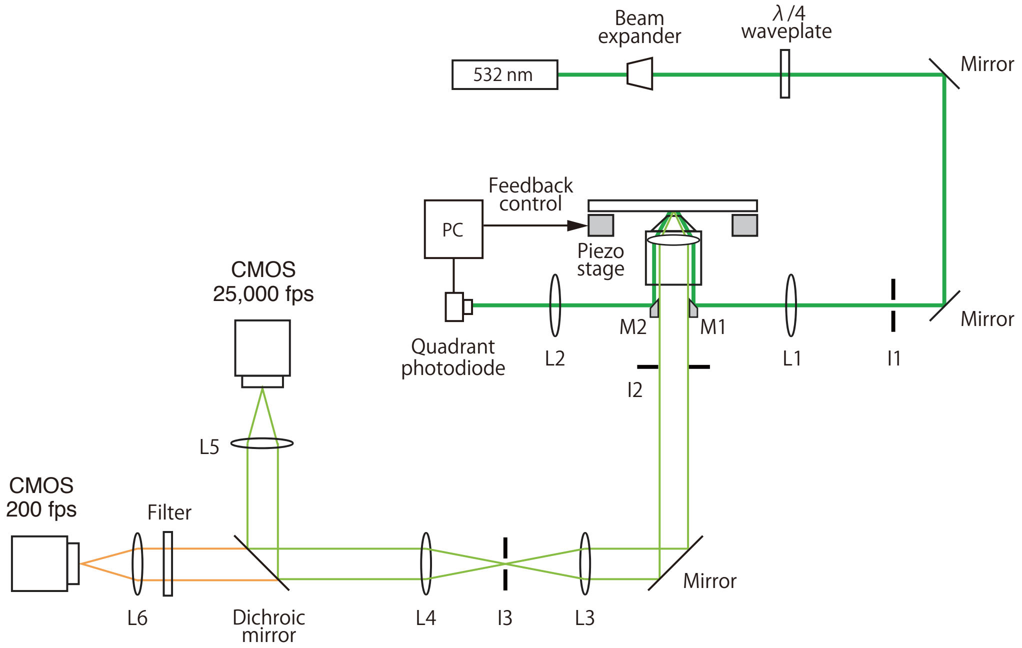

Supplementary Figure 3. A schematic of the TIRF setup. A 532 nm laser was collimated and expanded by a beam expander consisting of two lenses. A λ/4 waveplate was used to achieve circular polarized illumination. An iris (I1) was used to reduce the illumination area. The laser was focused onto the back focal plane of the objective by a lens (L1, 250 mm focal length) and micromirror (M1). The laser reflected on a glass surface immediately below the sample was directed into a quadrant photodiode by a micromirror (M2) and lens (L2) to perform feedback control of the piezo stage. An iris (I2) was used to attenuate scattering light. Scattering and fluorescent light were collimated by two lenses (L3 and L4), separated by a dichroic mirror and finally imaged onto CMOS cameras by imaging lenses (L5 and L6). An iris (I3) was used to define the field of view. A filter was used to increase the signal-to-noise ratio of the fluorescence image. See Methods for details.

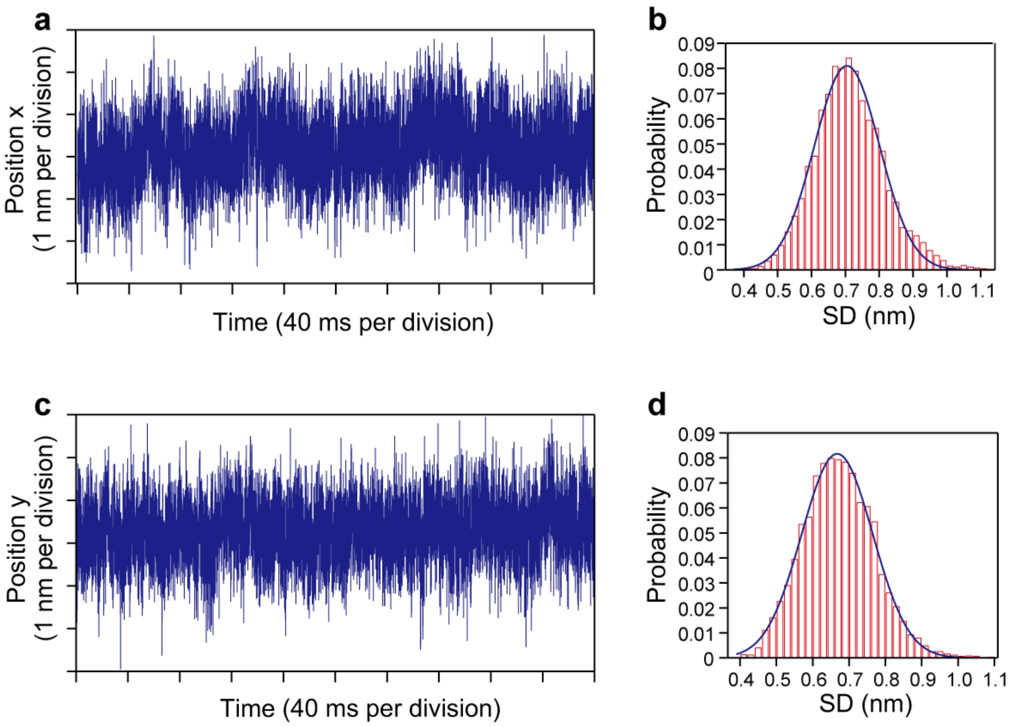

Supplementary Figure 4. A representative trace of an immobile GNP on a glass surface. An immobile GNP on a glass surface was imaged at 25,000 fps for 400 ms and localized. The drift of the stage was corrected by tracing a few immobile GNPs in the field of view, averaging the traces and subtracting from the raw trace. The GNP was immobilized on a glass surface via electrostatic interactions. **a**, **c** GNP trajectories in the x-direction (**a**) and y-direction (**c**). **b**, **d** Histograms of the standard deviations of the trajectories (**b**, x-direction; **d**, y-direction). The standard deviations were calculated every 25 points. The peaks are (x-direction) 0.70 ± 0.10 nm and (y-direction) 0.67 ± 0.10 nm (mean ± SD).

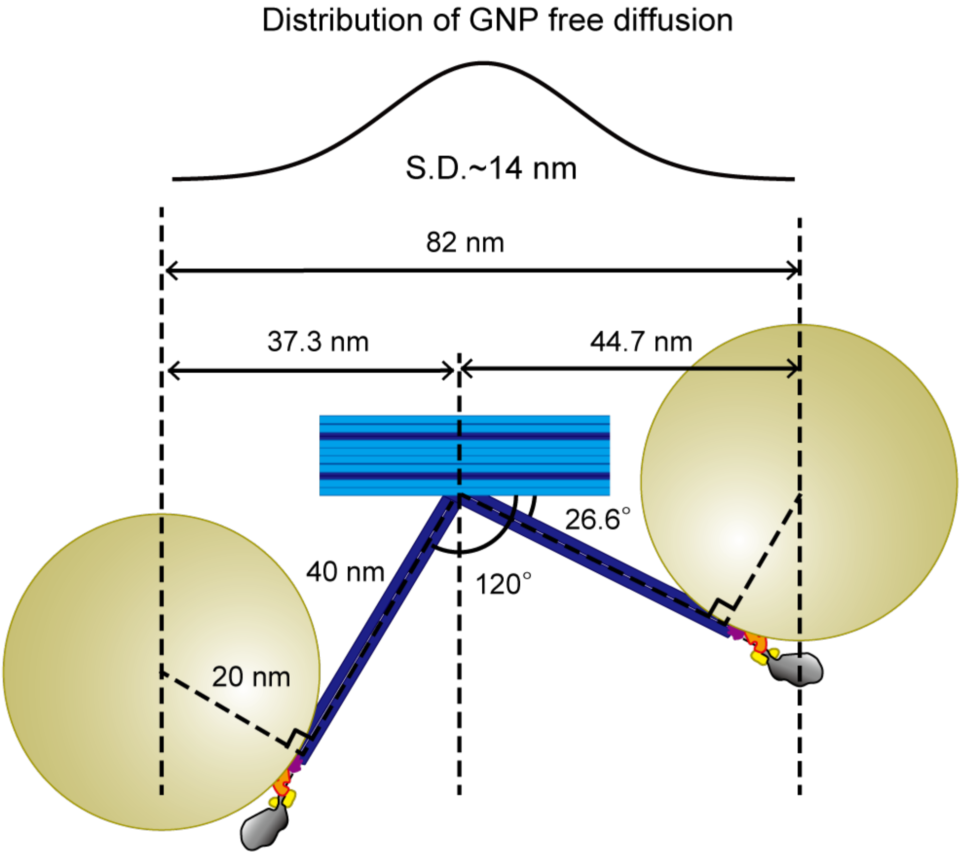

Supplementary Figure 5. A schematic geometry of the engineered thick filament-myosin-GNP complex. The range of free diffusion of a GNP attached to the engineered thick filament was restricted by the structure of the engineered thick filament. The right side is the direction of the lever-arm swing. The linker, which is 40 nm long, can pivot from 0 ° to 120 ° against the backbone (Fig. 1e). Consequently, the center of the GNP can freely diffuse from 26.6 ° to 120 ° on the assumption that it is rigidly attached to the end of the linker via bifunctional DNA linkers (Fig. 3b) and that S1 can flexibly rotate against the linker because of a nick in the DNA. The full range of GNP is 82 nm and, assuming a normal distribution of GNP free diffusion, the standard deviation is 14 nm. Our linker is approximately 3 times more flexible than native S2 (~20 nm peak-to-peak diffusion for native S2 tip[1] vs. ~60 nm peak-to-peak diffusion for our S2 tip).

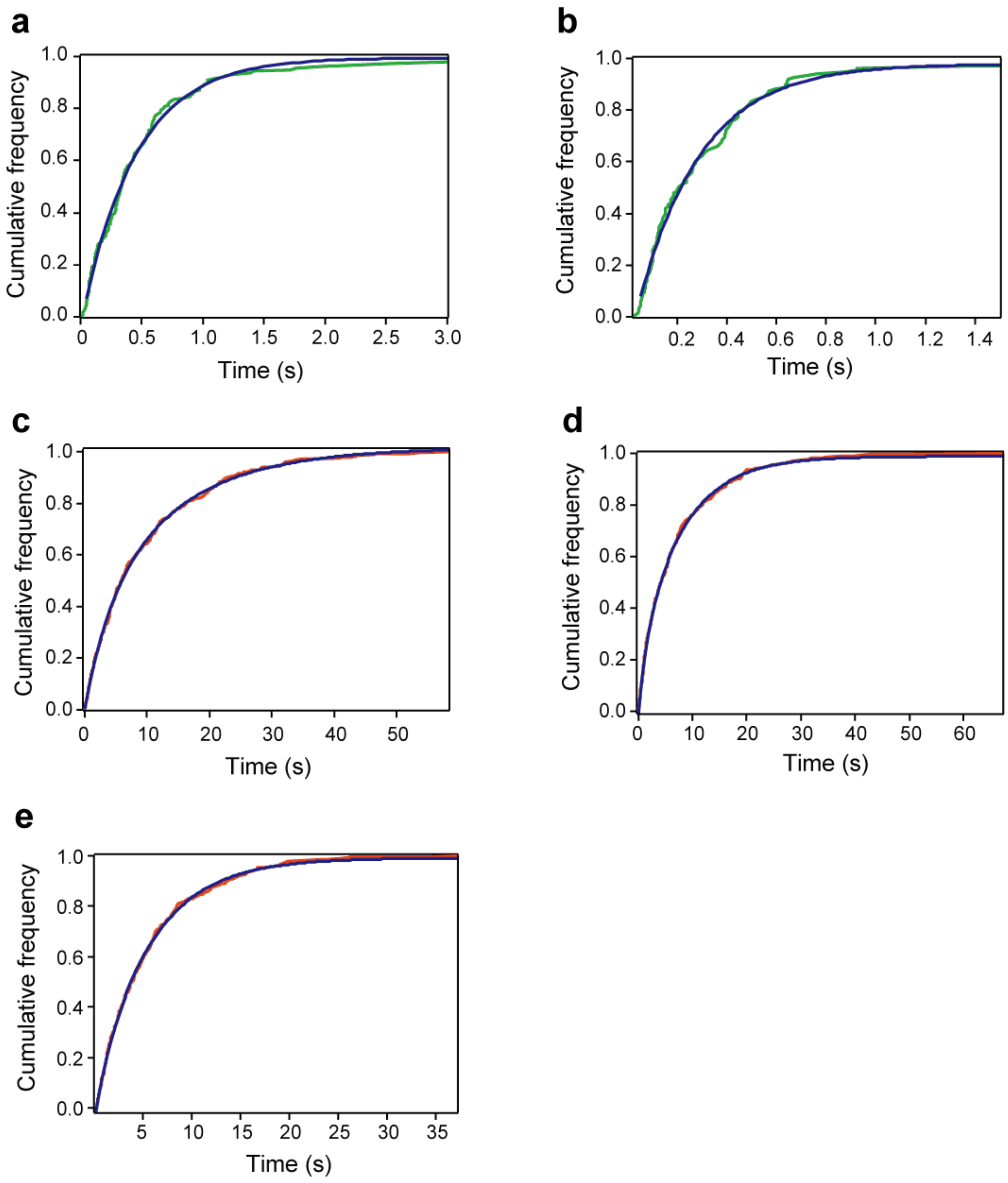

Supplementary Figure 6. Cumulative frequency plots of ATP waiting times. a, **b** Cumulative frequency of ATP waiting times at different ATP concentrations obtained by GNP tracking experiments. ATP waiting time corresponds to the binding times in Fig. 4a, c. Measurements of ATP waiting times were performed at (**a**) 500 nM (*n* = 177) and (**b**) 1000 nM ATP (*n* = 137). ATP waiting times were fit to the cumulative

distribution function of a single exponential decay (except those < 50 ms). The obtained decay rates were 2.25 ± 0.04 s^{-1} for **a** and 3.93 ± 0.09 s^{-1} for **b**. **c**-**e** Cumulative frequency of ATP waiting times at different ATP concentrations obtained by the fluorescent ATP experiments. Measurements of ATP waiting times were performed at (**c**) 25 nM (*n* = 409), (**d**) 50 nM (*n* = 435) and (**e**) 100 nM fluorescent ATP (*n* = 398). ATP waiting times were fit to the cumulative distribution function of a double exponential decay. The obtained decay rates were 0.25 ± 0.01 s^{-1} and 0.075 ± 0.003 s^{-1} for (**c**), 0.74 ± 0.03 s^{-1} and 0.127 ± 0.002 s^{-1} for (**d**), and 1.28 ± 0.21 s^{-1} and 0.183 ± 0.002 s^{-1} for (**e**).

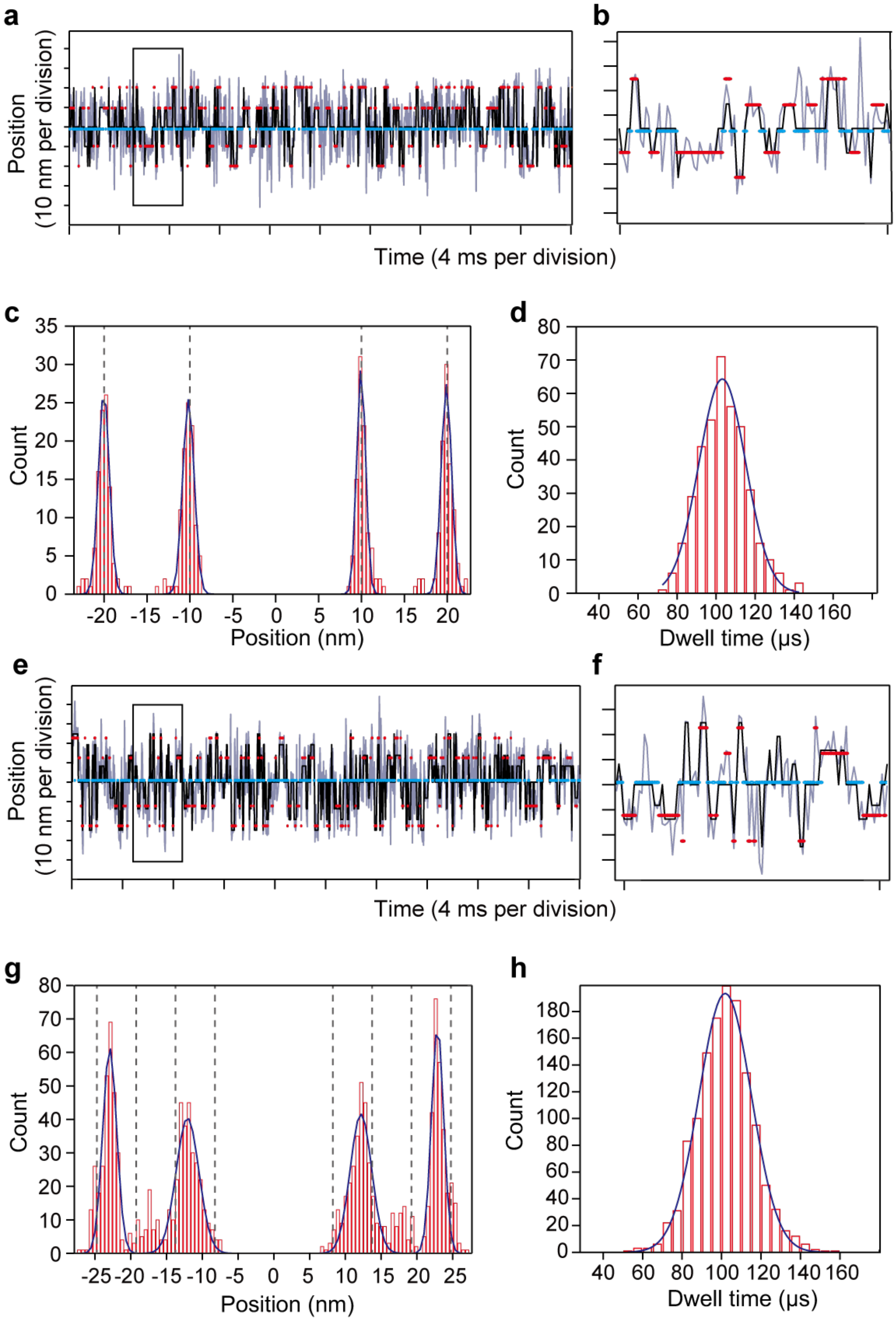
a
b
Position
(10 nm per division)
Time (4 ms per division)
c
d
Count
Position (nm)
Count
Dwell time (μs)
e
f
Position
(10 nm per division)
Time (4 ms per division)
g
h
Count
Position (nm)
Count
Dwell time (μs)

Supplementary Figure 7. Detection accuracy of nonparametric Bayesian inference. **a** Nonparametric Bayesian inference on a computationally produced test trajectory assuming 4 transient binding states whose binding positions are -20, -10, 10 and 20 nm (indicated by dashed lines), respectively, with a standard deviation of 4.7 nm, and a detached position at 0 nm with a standard deviation of 12.5 nm. The transition rate between the detached state and the binding states was set to 10,000 s^{-1}, which corresponds to a dwell time of 100 μs. A point was plotted every 40 μs. The black plot indicates the true state transition. Magenta and cyan plots indicate the inferred transient binding states and detached state, respectively. **b** An expanded trace of the box in (**a**). **c** A histogram of positions of the detected transient binding states. Positions were fit to four Gaussian distributions with peaks of -20.0 ± 0.6, -10.2 ± 0.7, 10.0 ± 0.5 and 19.9 ± 0.6 nm (mean ± SD). *n* = 628. **d** A histogram of the dwell times of the detected transient binding states. Dwell times were fit to a Gaussian distribution with a peak of 103.2 ± 12.0 μs (mean ± SD). *n* = 628. **e** Nonparametric Bayesian inference on a computationally produced test trajectory assuming eight transient binding states whose binding positions are -24.75, -19.25, -13.75, -8.25, 8.25, 13.75, 19.25 and 24.75 nm (indicated by dashed lines), respectively, the actin helical pitch, and a detached position at 0 nm. The standard deviations of the binding and detached states are the same as in **a**. **f** An expanded trace of the box in (**e**). **g** A histogram of positions of the detected transient binding states. Positions were fit to four Gaussian distributions with peaks of -22.9 ± 1.0, -12.1 ± 1.6, 12.2 ± 1.5 and 22.9 ± 0.9 nm (mean ± SD), respectively. *n* = 1310. **h** A histogram of dwell times of the detected transient binding states. Dwell times were fit to a Gaussian distribution with a peak at 101.8 ± 13.3 μs (mean ± SD). See also Methods. *n* = 1310.

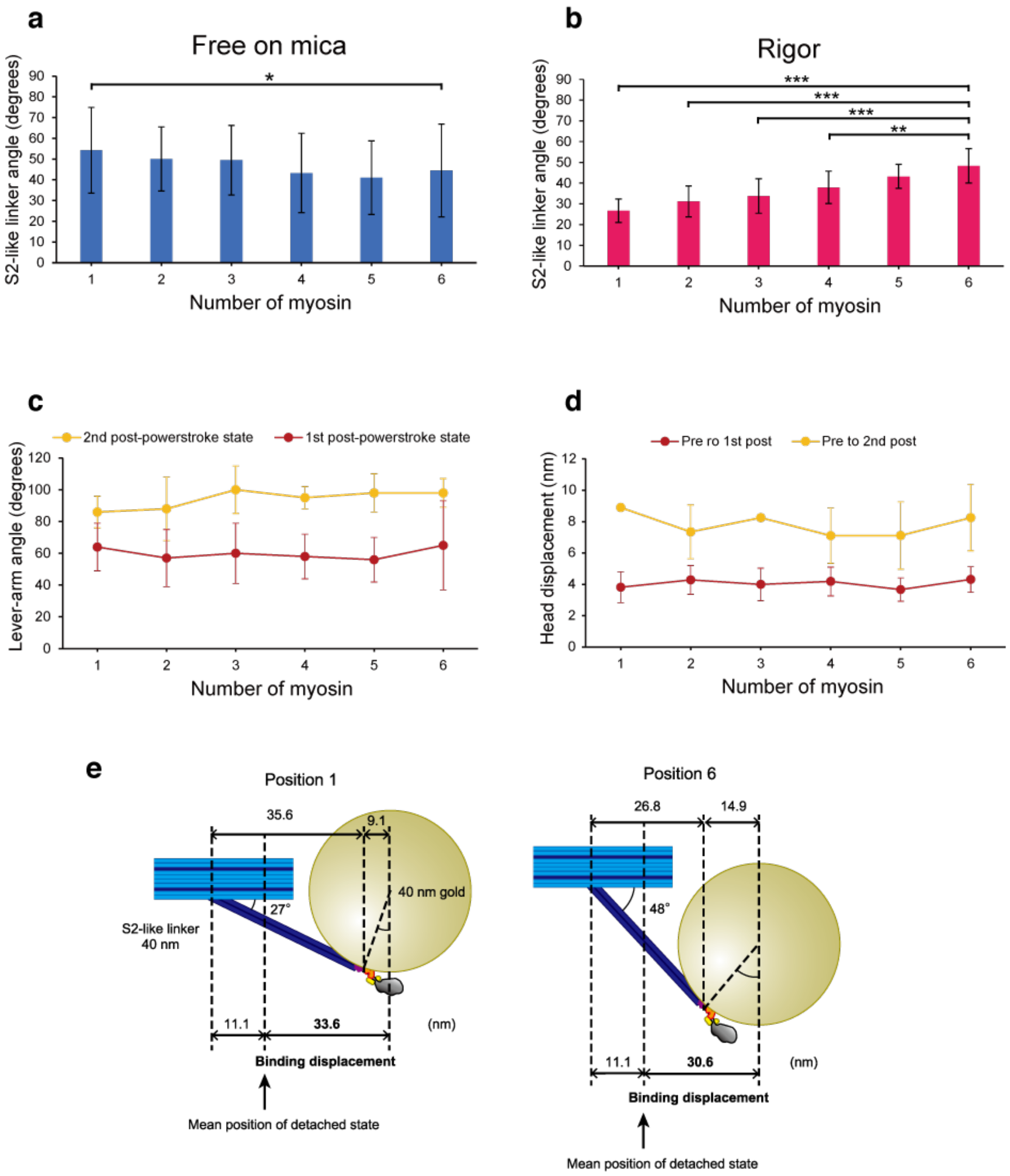

Supplementary Figure 8. Effects of linker angle on myosin motion. a Average linker angles at each myosin position in the free state on mica. $n = 47$ for myosin 1 and 51 for myosins 2-6 in the free state. Error bars indicate SD. $*p = 0.019$ (ANOVA with Dunnett's test). **b** Average linker angles at each myosin position in the rigor state on lipid. $n = 11$ for myosin 1, 18 for myosin 2 and 23 for myosins 3-6 in the rigor state. Error bars indicate SD. $**p = 0.0078$, $***p < 0.0001$ (ANOVA with Dunnett's test). **c**

Average lever-arm angles at the first and second post-powerstroke state at each myosin position. n = 22 for myosin 1, 58 for myosin 2, 87 for myosin 3, 99 for myosin 4, 66 for myosin 5 and 69 for myosin 6 at the first post-powerstroke state. n = 11 for myosin 1, 12 for myosin 2, 20 for myosin 3, 22 for myosin 4 and 23 for myosins 5-6 at the second post-powerstroke state. Error bars indicate SD. **d** Average head displacements from the pre- to first or second post-powerstroke state at each myosin position. n = 5 for myosin 1, 12 for myosin 2, 16 for myosins 3-4, 14 for myosin 5 and 13 for myosin 6 for the displacement from the pre- to first post-powerstroke state. n = 1 for myosin 1, 4 for myosin 2, 2 for myosin 3, 6 for myosins 4-5 and 9 for myosin 6 for the displacement from the pre- to second post-powerstroke state. Error bars indicate SD. **e** Position dependency of the binding displacement of GNP. The binding displacements at myosins 1 and 6 were calculated by the linker angles (**b**) and the binding displacement at position 3 (Fig. 4d), assuming that the mean position of the detached state is unchanged by the linker positions. The estimated binding displacements (33.6 nm vs. 33.3 nm vs. 30.6 nm at myosin 1, 3 and 6, respectively) suggest that the position dependency of the angle has a negligible effect on the displacement.

Supplementary Reference

1 Kaya, M. & Higuchi, H. Nonlinear elasticity and an 8-nm working stroke of single myosin molecules in myofilaments. *Science* **329**, 686-689 (2010).
