# Supplementary material for: Direct visualization of human myosin II force generation using DNA origami-based thick filaments
Source: Commun Biol. 2019 Nov 27;2:437. doi: 10.1038/s42003-019-0683-0 (PMC6881340; doi:10.1038/s42003-019-0683-0)
Supplement: Supplementary file 2 — Description of Additional Supplementary Files [file 42003_2019_683_MOESM2_ESM.pdf]

Supplementary Movie 1. High-speed AFM movie showing the two-step lever-arm swing of myosin II.

The dynamic process at 190 nM was filmed at 400 ms $frame^{-1}$ (2.5 frames s^{-1}). Orientation changes in myosin II S1 were observed in a two-step manner. Image area, 66 × 96 nm^2 with 220 × 320 pixels (magnified 10 times for clarity by image processing).

Supplementary Movie 2. High-speed AFM movie showing reversed the lever-arm swing of myosin II.

The dynamic process at 190 nM caged ATP was filmed at 400 ms $frame^{-1}$ (2.5 frames s^{-1}). Image area, 66 × 81 nm^2 with 220 × 270 pixels (magnified 10 times for clarity by image processing).

Supplementary Data 1. Source data.

All source data in the main figures are available in Supplementary Data 1.

Supplementary Data 2. Oligonucleotides sequences for the DNA rod.

This file contains the sequences of the core staples used to build the DNA rod for the synthetic thick filament and the sequences of the handle staples attached to myosin and the actin binding domain of α-actinin.
